# Lactose intolerance and probiotics: from pathophysiological mechanisms to clinical applications

**DOI:** 10.1007/s10482-026-02278-x

**Published:** 2026-03-17

**Authors:** Tsachi Tsadok Perets, Rachel Gingold-Belfer, Ram Dickman

**Affiliations:** 1 The Gastroenterology Laboratory and the Division of Gastroenterology, Rabin Medical Center, Beilinson Campus, Petach Tikva, Israel; 2 Department of Digital Medical Technologies, Holon Institute of Technology, Holon, Israel; 3 Gray School of Medicine, Tel Aviv University, Tel Aviv, Israel

**Keywords:** Lactase deficiency, GI probiotics, Microbiota, Beta-galactosidase, Fermented dairy

## Abstract

Lactose is a disaccharide found in dairy products, which provide energy and essential nutrients. Digestion of lactose relies on the intestinal enzyme lactase, or lactase-phlorizin hydrolase, located on the brush border of the small bowel mucosa. This enzyme splits lactose into two absorbable monosaccharides: glucose and galactose. When lactase activity is insufficient, undigested lactose proceeds to the colon where it is fermented by the gut flora, generating gas that trigger the uncomfortable symptoms associated with lactose intolerance. Lactase non-persistence is extremely common, affecting approximately 70% of the adult population world-wide. Prevalence varies markedly across geographic regions, typically ranging from 50 to 90% in African, Asian, and South American countries. The subjective diagnosis of lactose intolerance requires the occurrence of symptoms such as abdominal pain, bloating, flatulence and diarrhea following the ingestion of high lactose dairy products. An objective assessment of lactose intolerance may be achieved with a specific breath test that measures hydrogen emission in breath following the ingestion of lactose. Consequently, current international guidelines require concurrent report of typical symptoms and pathologic breath test results in order to diagnose lactose intolerance. Management of lactose intolerance often involves dietary restrictions and the prescription of formulations that contain lactase. However, one should recognize that avoiding dairy products can increase the risk of nutritional deficiencies. Therefore, ongoing research is focused on alternative strategies, notably utilizing gut microbiota in order to improve tolerance to lactose. This review aims to explore the evidence supporting the use of probiotics as a potential treatment strategy to alleviate the symptoms of lactose intolerance by modulating colonic metabolism and enhancing lactose digestion.

## Methodology

This narrative review summarizes pathophysiology and clinical evidence on probiotics for lactose intolerance. We prioritized human interventional studies and meta-analyses reporting symptom outcomes during lactose exposure and/or breath-test endpoints. Table 1 highlights probiotic strains/products supported by controlled clinical studies in people with lactose malabsorption.

**Table 1:** Evidence based probiotic strains useful in LI (controlled human trials in lactose maldigesters reporting symptom outcomes and/or breath-test endpoints)

Strain	Evidence strength	Proposed Mechanism
**Lactobacillus acidophilus DDS-1 (**Pakdaman et al. 2016**)**	Randomized, double-blind, placebo-controlled crossover trial reported reduced lactose intolerance symptom scores after supplementation, with improved tolerance to lactose challenge	High beta-galactosidase (lactase) activity enhances lactose hydrolysis; may reduce colonic lactose load and gas production; potential modulation of gut microbiota
**Bifidobacterium animalis subsp. lactis Bi-07 (**Rasinkangas et al. 2022**)**	Demonstrated support for lactose digestion in vitro and in randomized placebo- and lactase-controlled clinical trials; associated with reduced gastrointestinal symptoms during lactose exposure	beta-galactosidase delivery to the small intestine and enhanced lactose breakdown; may shift fermentation toward less gas-producing pathways
**Multi-strain probiotic formulation (Bio-25) (**Gingold-Belfer et al. 2020**)**	Pilot study in lactose-intolerant adults reported reduced symptom severity and improved quality-of-life measures following a multi-strain probiotic intervention	Combined lactase-positive strains increase overall beta-galactosidase capacity; possible community-level effects (cross-feeding, SCFA production) that improve tolerance
**Bifidobacterium longum BB536 + Lactobacillus rhamnosus HN001 (fermented dairy) (**Pakdaman et al. 2016**)**	Randomized, double-blind, cross-over study in lactose-intolerant subjects showed symptom improvement and favorable breath-test responses when consuming fermented dairy supplemented with these probiotics	Enhanced lactose digestion via microbial beta-galactosidase; modulation of microbial fermentation and visceral sensitivity; potential barrier and immune effects
**Bifidobacterium animalis subsp. animalis IM386 + Lactiplantibacillus plantarum MP2026 (**Jaagura et al. 2022**)**	Placebo-controlled clinical trial reported improved lactose tolerance and reductions in symptoms during lactose challenge after probiotic supplementation	Provision of lactase activity plus possible effects on intestinal motility and fermentation patterns, reducing gas and osmotic load
**Probiotic yogurt fortified with Lactobacillus acidophilus and Bifidobacterium sp. (**Roškar et al. 2017**)**	Clinical intervention with fortified probiotic yogurt improved self-reported gastrointestinal symptoms and markers of lactose tolerance compared with control dairy	Starter and added cultures contribute beta-galactosidase; dairy matrix improves survival and in situ lactose hydrolysis; may promote colonic adaptation
**Ice cream containing Bifidobacterium bifidum 900,791 (**Vitellio et al. 2019**)**	Hypolactasic subjects consuming probiotic ice cream showed improved lactose tolerance measures compared with control products	Cold dairy matrix can deliver viable bacteria and beta-galactosidase; slower gastric emptying may facilitate lactose digestion; may alter colonic fermentation
**Limosilactobacillus reuteri DSM 17938 (**Leis et al. 2020; Masoumi et al. 2021**)**	Included among probiotic interventions evaluated in controlled-trial systematic reviews/meta-analyses; evidence suggests potential symptom benefit in some cohorts	Potential lactase activity and effects on gut motility and mucosal environment; may reduce inflammatory signaling and improve barrier function
**Lacticaseibacillus casei Shirota + Bifidobacterium breve (various preparations) (**Ahn et al. 2023; Almeida et al. 2012**)**	Evaluated across controlled trials summarized in systematic reviews/meta-analyses; some studies report symptom relief and improved breath-test profiles	Likely driven by microbial beta-galactosidase and shifts in colonic fermentation; possible modulation of sensory signaling contributing to symptom reduction
**Yogurt starter cultures: Streptococcus thermophilus and Lactobacillus delbrueckii subsp. bulgaricus (**Savaiano and Hutkins 2021; Leis et al. 2020; Ahn et al. 2023; Oak and Jha 2019; Liao et al. 2022**)**	Live-culture yogurt and fermented dairy are associated with improved lactose digestion and tolerance in lactose maldigesters; benefits supported by clinical and review literature	Starter cultures supply beta-galactosidase and pre-digest lactose; viscous dairy matrix slows transit and improves lactose digestion; may support microbiome adaptation
**Bifidobacterium-containing fermented dairy (various B. longum/B. animalis strains) (**Ahn et al. 2023; Roškar et al. 2017; Oliveira et al. 2022**)**	Across trials and meta-analyses, Bifidobacterium-containing fermented dairy products show overall symptom improvement trends in lactose-intolerant adults, though effects are strain- and product-dependent	Increased luminal beta-galactosidase and cross-feeding to SCFA producers; potential reduction of hydrogen production and improved epithelial tolerance

Searches were conducted using targeted queries in major biomedical databases (e.g., PubMed/MEDLINE) and complementary search engines (e.g., Google Scholar), combining terms related to lactose maldigestion/intolerance ("lactose intolerance", "lactase non-persistence", "hydrogen breath test") with probiotic and fermented dairy terms ("probiotic", "Lactobacillus", "Bifidobacterium", "beta-galactosidase", "yogurt", "fermented milk"). Reference lists of relevant reviews, guidelines, and key clinical trials were also screened to identify additional studies. Priority was given to human randomized or controlled trials and meta-analyses reporting symptom outcomes during lactose exposure and/or objective breath-test measures. Mechanistic and observational studies were used to support background concepts when clinical evidence was limited; no formal systematic review protocol or meta-analysis was performed.

## Introduction—pathophysiology of lactose intolerance

Lactose intolerance (LI) is a clinical syndrome that results from lactose maldigestion and malabsorption (LM). The basic cause of LI is an insufficient level of small intestine—brush border lactase, lactase-phlorizin hydrolase (LPH) activity (Misselwitz et al. 2019). Lactase is the only enzyme able to cleave disaccharide such as lactose into the absorbable monosaccharides, glucose and galactose. Whenever there is insufficient level of lactase, undigested lactose reaches the colon and undergoes microbial fermentation (Misselwitz et al. 2019; Fassio et al. 2018; Rezaie et al. 2017). Lack of sufficient amount of lactase can be caused by different factors, leading to three or four main types of lactase deficiency (LD) (Misselwitz et al. 2019; Fassio et al. 2018; Rezaie et al. 2017; Deng et al. 2015; Szilagyi and Ishayek 2018).

1. Primary lactase deficiency – Adult-Type Hypolactasia or Lactase Non-Persistence (LNP), that is the most common cause of LM in adolescents and adults and is genetically determined (Misselwitz et al. 2019; Fassio et al. 2018; Rezaie et al. 2017). This entity is characterized by a progressive and permanent decrease in intestinal—brush border lactase expression. It starts typically in late childhood (at the age of 5–6 years in European and American descendants) or adolescence, though it can begin even earlier (at the age of 2–3 years) in children of African, Asian or Hispanic descent (Misselwitz et al. 2019; Rezaie et al. 2017; Deng et al. 2015; Bouchoucha et al. 2021). This genetically programmed down-regulation is considered the biological wild-type condition, not a disease. The ability of lactase to persist into adulthood (lactase persistence) is inherited as a dominant trait and is regulated by specific single nucleotide variants (SNPs) in a regulatory region upstream of the lactase gene (LCT) on chromosome number 2. The LNP genotype leads to the natural decline of lactase expression (Misselwitz et al. 2019; Deng et al. 2015; Bouchoucha et al. 2021).
2. Congenital Lactase Deficiency (CLD) is an extremely rare and severe autosomal recessive disease characterized by the total absence or significant reduction of lactase activity from birth. Symptoms, such as watery diarrhea and malnutrition, begin immediately with the consumption of breast milk or lactose-containing formulas. This condition is caused by severe mutations in the gene itself (Deng et al. 2015; Vesa et al. 2000).
3. Secondary Lactase Deficiency (Secondary Hypolactasia) is a transient lack of lactase due to divers structural or inflammatory processes that damage the epithelium or the small intestinal villi. Common etiologies include celiac disease, Crohn’s disease, infectious gastroenteritis (e.g., Rotavirus gastroenteritis or parasitic diseases like Giardia), or even damage from radiation therapy or chemotherapy. In this entity loss of lactase activity is usually reversible, improving once the underlying intestinal injury or disorder is successfully treated (Misselwitz et al. 2019; Rezaie et al. 2017; Bouchoucha et al. 2021; Vesa et al. 2000).

## Epidemiology and public health impact

LM and LI are extremely common conditions, affecting the majority of the adult population. Approximately 70% of the adult population worldwide is considered to be LNP (Szilagyi and Ishayek 2018; Bouchoucha et al. 2021; Vesa et al. 2000). The real prevalence of confirmed LI cases worldwide is estimated to exceed 65%, while the prevalence of confirmed cases is around 57%. The prevalence of LNP shows significant variability across geographic regions and ethnic groups. High prevalence regions include continents and countries where dairy farming was not historically widespread. For example, in Asia, the prevalence of LNP is extremely high, exceeding 90% in some countries and up to 100% in others. In Africa, the prevalence of LNP is also very high, ranging from 65 to 75% up to almost 100% in some regions. In South America and Latin American countries, the prevalence of LNP is reported to be over 50% (Fassio et al. 2018; Vesa et al. 2000).

Moderate/low prevalence regions include populations in northern central Europe where LNP is reported to be low (2 to 20%). For example, in Denmark and other Scandinavian countries, the prevalence may reach 5%. In southern Europe (mediterranean countries), the prevalence is higher, around 40 to 70% (Southern Italy). In the United States the prevalence of LNP varies significantly based on the ethnic origin. The lowest prevalence is found in European origin communities (15–20%), while it is much higher in Mexican-Americans/Hispanics (53%) and in Afro-American communities (60–80%) (Fassio et al. 2018; Bouchoucha et al. 2021; Vesa et al. 2000).

Historically, this geographical diversity can be partially explained by the gene-culture-coevolution hypothesis, suggesting that populations with a background of domesticating and consuming mammalian milk developed lactase persistence (LP), which spread through selection pressure, particularly in northern European regions where dairy products became an important part of the diet (Vesa et al. 2000; Hodges et al. 2019).

LI has important dietary and cultural implications as it significantly impacts the lives of affected individuals and leads to changes in eating habits. Dairy avoidance is the most common practice of individuals who experience LI symptoms or perceive themselves to be intolerant (Deng et al. 2015; Vesa et al. 2000; Hodges et al. 2019). These individuals favorably respond to reduction or elimination of milk and many dairy products. This avoidance can begin early in life, sometimes unnecessarily, even interrupting breastfeeding or generating a habit toward dairy elimination in children and adolescents (Hammer et al. 2022). Low dairy consumption is particularly noted in populations with a high LNP prevalence including Asian immigrants to the United States, suggesting that milk drinking habits are influenced by genetics and culture (Hodges et al. 2019; Hammer et al. 2022).

The avoidance of dairy foods has a significant public health impact such as nutrient deficiencies. Dairy products are excellent sources of essential nutrients including calcium, potassium, vitamin D, B vitamins, and high-quality protein. Cow’s milk is also a major source of phosphorus, choline, riboflavin, and vitamin B12 (Fassio et al. 2018; Hodges et al. 2019; Hammer et al. 2022). Thus, when dairy products are completely eliminated from the diet, individuals, especially children and adolescents, may face an increased risk of nutritional deficiencies and other adverse health outcomes, including bone fracture and osteoporosis (Fassio et al. 2018; Bouchoucha et al. 2021; Hodges et al. 2019; Hammer et al. 2022). The complete withdrawal from milk products for a prolonged period is a potential risk factor for defective bone mineralization. Low dietary milk and dairy intake have been identified as a risk factor for bone fracture and osteoporosis. Studies show that lactose intolerant subjects consume lower amounts of calcium compared to tolerant people, with average intake often falling below the Recommended Dietary Allowance (RDA) of 1000 mg/day for adults (Fassio et al. 2018; Deng et al. 2015; Hodges et al. 2019; Hammer et al. 2022). Global evaluations show that in Asian countries (high LNP), consumption is generally low (< 500 mg/day), while in northern Europe (low LNP) it is above 1000 mg/day. Low calcium consumption increases the risk for chronic diseases, most notably osteoporosis and its sequelae. It is important to underline that if dairy products are eliminated, other dietary sources of calcium or calcium supplements need to be provided to meet recommended intake levels (e.g., 1,300 mg/day for individuals over 10 years, based on EFSA guidelines) (Fassio et al. 2018; Deng et al. 2015; Bouchoucha et al. 2021; Hodges et al. 2019; Hammer et al. 2022). Interestingly, lactose itself enhances the absorption of calcium. As a result, the dairy industry has made big efforts to minimize the influence of LI on milk consumption. Educational efforts now focus on encouraging the consumption of tolerable amounts of milk, often ingested with meals to delay gastric emptying and dilute lactose, or the use of lowered lactose-containing foods such as hard cheeses, yogurt, and lactose-hydrolyzed milk products. Fermented dairy products, such as yogurt and hard cheeses, have long been employed as a strategy for overcoming LI because they contain lactose that is partially digested by live bacteria. Furthermore, milk consumption during early life is important for normal growth, as it provides energy, high-value proteins, vitamins and calcium (Fassio et al. 2018; Hodges et al. 2019; Montalto et al. 2005). However, older adults are another important target as inadequate dietary intake (due to LI) in this population carries greater detrimental impact especially due to osteoporosis and falling risk. Moreover, the costs associated with over-the-counter lactase supplements can be high (Montalto et al. 2005; Ianiro et al. 2016).

Once undigested lactose reaches the large intestine, resident gut microbiota readily ferment it (Fassio et al. 2018; Bouchoucha et al. 2021; Montalto et al. 2005). This bacterial fermentation produces volatile fatty acids, including short-chain fatty acids (SCFA) and various gases, primarily hydrogen (H_2_), carbon dioxide (CO_2_), methane (CH_4_) and hydrogen sulfide (H_2_S) (Misselwitz et al. 2019; Fassio et al. 2018; Deng et al. 2015). The rapid production and accumulation of these gases is responsible for bloating, distention, abdominal pain and even constipation (Montalto et al. 2005; Ianiro et al. 2016; Ibba et al. 2014). Typical symptoms associated with LI usually develop within 30 min to 2 h after the ingestion of lactose-containing foods. Gas production leads to distension of the small bowel and increased intraluminal pressure, resulting in abdominal pain, cramping, intestinal rumbling, bloating, and flatulence (Harvey et al. 2018; Ruzsanyi et al. 2016; Baijal and Tandon 2020). The severity of these symptoms depends heavily on individual visceral sensitivity to distension and in patients with irritable bowel syndrome (IBS) often experience more severe symptoms than controls (Misselwitz et al. 2019; Fassio et al. 2018; Hodges et al. 2019; Montalto et al. 2005; Harvey et al. 2018).

Diarrhea may result from an excessive osmotic load exerted by non-digested lactose in the lumen (Hammer et al. 2022; Montalto et al. 2005; Binder 2010; Read 1982). The osmotic force drives water and electrolytes into the gut lumen, resulting in watery and sometimes frothy stools. Uncommon symptoms include nausea, vomiting, headache (fogginess), vertigo, memory impairment, lethargy, muscle/joint pain, or mouth ulcers, which may be caused by toxic metabolites that can alter cell-signaling mechanisms (Misselwitz et al. 2019; Deng et al. 2015; Hammer et al. 2022; Baijal and Tandon 2020; Campbell et al. 2010).

Diagnosis is typically based on symptoms after lactose ingestion supported by objective testing. Hydrogen/methane breath testing remains the most widely used noninvasive test, while genetic testing for lactase persistence and point-of-care lactase activity assays (e.g., Lactose Quick Test) may be helpful in selected cases (Rezaie et al. 2017; Hammer et al. 2022; Ruzsanyi et al. 2016; Baijal and Tandon 2020).

## Management—The conventional approaches

Fermented dairy (especially yogurt with live cultures) can improve lactose digestion compared with unfermented milk because starter cultures provide beta-galactosidase activity and slow gastric emptying, allowing many lactose-intolerant individuals to tolerate modest lactose loads. This is particularly relevant when balancing symptom control with adequate calcium/vitamin D intake (Montalto et al. 2005; Ianiro et al. 2016; Savaiano and Hutkins 2021).

In humanitarian and pediatric nutrition contexts, milk lactose remains an important carbohydrate source in therapeutic foods; clinical decisions should weigh intolerance symptoms against nutritional needs, especially in undernourished children (Harvey et al. 2018; Baijal and Tandon 2020).

The traditional management of LI focuses on reducing or eliminating the consumption of lactose-containing foods to alleviate symptoms. The mainstay of treatment is a low-lactose diet, although complete dairy avoidance is generally no longer recommended for most individuals (Rezaie et al. 2017). Available data suggest that adolescents and adults can typically ingest up to 12 to 15 g of lactose in a single dose (equivalent to about one cup of milk) with minimal or no symptoms, particularly if consumed alongside a meal to slow transit time (Ibba et al. 2014; Baijal and Tandon 2020; Savaiano and Hutkins 2021). Dietary modifications encourage the consumption of dairy products naturally low in lactose, such as certain hard cheeses and yogurts, where the lactose has been partially predigested by live bacteria (Savaiano and Hutkins 2021). Furthermore, the food industry offers lactose-free and lactose-reduced products, achieved by adding exogenous lactase (beta-galactosidase) to hydrolyze the lactose into glucose and galactose prior to consumption. Plant-based dairy alternatives, such as soy, almond, or oat milk, are another option, and these are frequently fortified with essential nutrients. Another conventional strategy is lactase enzyme replacement therapy, which involves administering exogenous lactase, derived typically from fungi or yeast, in order to correct the native lactase deficiency (Montalto et al. 2005; Ianiro et al. 2016; Ibba et al. 2014). Nevertheless, and despite these established approaches the risk of nutritional deficiencies (calcium and vitamin D) is not completely eliminated. Dairy foods are excellent sources of essential nutrients, and the complete withdrawal from milk products for prolonged periods poses an increased risk for adverse health outcomes like bone fracture and osteoporosis (Szilagyi and Ishayek 2018; Hodges et al. 2019; Harvey et al. 2018). Consequently, individuals who eliminate dairy must ensure they receive adequate calcium intake (e.g., 1,300 mg/day for individuals over 10 years old) through alternative foods or supplements (Misselwitz et al. 2019; Szilagyi and Ishayek 2018; Hodges et al. 2019; Ibba et al. 2014). Moreover, adherence to a strictly lactose-free diet is challenging due to the pervasive use of “hidden lactose” as an additive in many processed foods, baked goods, and even medications. For many individuals, the small amounts of lactose used as pharmaceutical excipients are well tolerated; however, it may be relevant in highly sensitive individuals or with cumulative exposure (Eadala et al. 2009). In terms of enzyme replacement, the efficacy of oral lactase supplements is widely reported as modest and variable among patients (Szilagyi and Ishayek 2018; Hodges et al. 2019; Ruzsanyi et al. 2016). The supplement’s effect is short-lived, requiring timely consumption just before the meal (Ianiro et al. 2016; Ibba et al. 2014). Furthermore, assessing the exact concentration of lactase needed to fully digest the lactose in a particular dairy product is technically difficult, and the commercial supplements can be costly (Fassio et al. 2018; Rezaie et al. 2017; Montalto et al. 2005). These limitations highlight the ongoing need for alternative, effective strategies, particularly those that address lactose maldigestion internally, such as modulating the colonic microbiota (Misselwitz et al. 2019; Fassio et al. 2018; Szilagyi and Ishayek 2018; Hodges et al. 2019; Montalto et al. 2005; Ianiro et al. 2016; Savaiano and Hutkins 2021; Kato et al. 2018).

## Gut microbiota & lactose metabolism

Undigested and absorbed lactose, due to lactase deficiency, reaches the colon and undergoes colonic fermentation by gut microbiota. The digestion of lactose is completed by microbial lactase such as beta-galactosidase (Misselwitz et al. 2019; Schmidt et al. 2020). This metabolic process also known as fermentation leads to the production of SCFA such as acetate, propionate, butyrate, lactate and various gases, predominantly H_2_ and CO_2_, and to a lesser extent CH_4_ or H_2_S (JanssenDuijghuijsen et al. 2024). Gas type depends on the microbial composition within the colon. Certain bacteria, particularly species within the Bifidobacterium and Lactobacillus genera, are capable of digesting and utilizing lactose for energy via their bacterial lactase (Kato et al. 2018; JanssenDuijghuijsen et al. 2024). In addition, these specific bacteria also produce SCFAs, important energy sources for colonic epithelial cells. Interestingly, Bifidobacterium and Lactobacillus often do not produce gases like hydrogen and methane, unlike other heterofermentative bacteria in the colon (Misselwitz et al. 2019; Fassio et al. 2018; Schmidt et al. 2020; Bonder et al. 2016; Kurilshikov et al. 2021).

Another important observation in LI is the phenomenon of microbiota adaptation that results in the development of tolerance to lactose (JanssenDuijghuijsen et al. 2024; Bonder et al. 2016; Kurilshikov et al. 2021; Vitellio et al. 2019). Microbiota adaptation refers to changes in microbial composition and metabolic activity in response to regular or chronic consumption of lactose, leading to clinical tolerance: less symptoms and normal breath hydrogen excretion. Microbial beneficial changes include proliferation and abundance of lactose-fermenting microorganisms like Bifidobacteria and Lactobacilli that possess beta-galactosidase activity (JanssenDuijghuijsen et al. 2024; Bonder et al. 2016; Kurilshikov et al. 2021). These metabolic changes result in a better digestion of lactose in the colon and enhanced utilization of hydrogen produced during fermentation. Finally, colonic adaptation to lactose intake is generally a reversible process. If lactose is eliminated from the diet, the adaptation can dissipate gradually, potentially causing symptoms again upon re-introduction of lactose. This transient nature highlights that adaptation relies on sustained lactose exposure, rather than an upregulation of the innate human lactase enzyme in the small intestine (Schmidt et al. 2020; Bonder et al. 2016; Kurilshikov et al. 2021; Vitellio et al. 2019). Because lactose that reaches the colon can selectively support lactose-fermenting taxa (e.g., Bifidobacterium), regular low-to-moderate lactose exposure may have prebiotic-like effects and contribute to adaptation. Related disaccharides such as lactulose have also been explored as microbiota-modulating substrates, although their clinical role in lactose intolerance remains less well defined.

Modulation of colonic microbiota may be related to an interaction with the host genome, particularly with the LCT gene region and may be achieved using probiotics supplements (Bonder et al. 2016; Kurilshikov et al. 2021; Vitellio et al. 2019).

## Probiotics: definition and mechanism of action in LI

Probiotics are defined as live microorganisms that, when administered in adequate amounts, confer health benefits to the host (Hill et al. 2014). Probiotics typically belong to the genera Lactobacillus and Bifidobacterium, although other bacteria and certain yeasts like Saccharomyces boulardii may also have probiotic properties (Hill et al. 2014; Gibson et al. 2017). Probiotics can modulate intestinal microbial activity and host responses (Hill et al. 2014; Gibson et al. 2017; Salminen et al. 2021). Furthermore, it has been proposed that probiotics, particularly strains capable of expressing beta-galactosidase activity may become a valid therapeutic option in LI. Production and delivery of beta-galactosidase to gut lumen is the principal mechanism by which probiotics may alleviate symptoms related to LI (Leis et al. 2020).

After consumed, galactosidase-producing probiotic strains survive the passage through the stomach and hydrolyze undigested lactose in the small intestine. It is particularly effective when probiotics are consumed as part of fermented milk products like yogurt, which already contain microbial galactosidase (Szilagyi and Ishayek 2018; Savaiano and Hutkins 2021; Leis et al. 2020; Ahn et al. 2023). Some studies suggest that during transit in the gastrointestinal tract, strains as Bifidobacterium and Lactobacillus may be lysed by bile, that result in the releasing of galactosidase into the gut lumen (Szilagyi and Ishayek 2018; Bouchoucha et al. 2021; Ahn et al. 2023). Other studies suggest that probiotics can transfer lactase outside their cell membranes. The net result of galactosidase-producing probiotic strains is the reduction of the amount of lactose that may reach the colon and the production of fermentation byproducts as H_2_, CO_2_, CH_4_, H_2_S and SCFAs (Szilagyi and Ishayek 2018; Bouchoucha et al. 2021; Leis et al. 2020; Ahn et al. 2023; Rasinkangas et al. 2022). Accordingly, some clinical benefit may derive from delivery of microbial beta-galactosidase activity even when viability is reduced during transit, conceptually overlapping with postbiotic approaches (Leis et al. 2020).

Another beneficial effect of probiotic administration is modulation of composition and metabolic activities of colonic microbiota. Consumption of probiotics such as Bifidobacterium longum and Lactobacillus acidophilus can support the growth and colonization of Bifidobacterium animalis, and increase beta-galactosidase activity (Leis et al. 2020; Angima et al. 2024; Gingold-Belfer et al. 2020). It is noteworthy that the majority of probiotic strains appear to be transient and do not permanently colonize the adult gut (Leis et al. 2020; Angima et al. 2024; Gingold-Belfer et al. 2020; Berstad et al. 2016).

Furthermore, probiotics may suppress heterofermentative bacteria responsible for gas production (hydrogen and methane). This may be achieved by the secretion of antimicrobial substances like bacteriocins or organic acids and by competitive adherence to the mucosa (Angima et al. 2024; Gingold-Belfer et al. 2020).

**Fig. 1:**
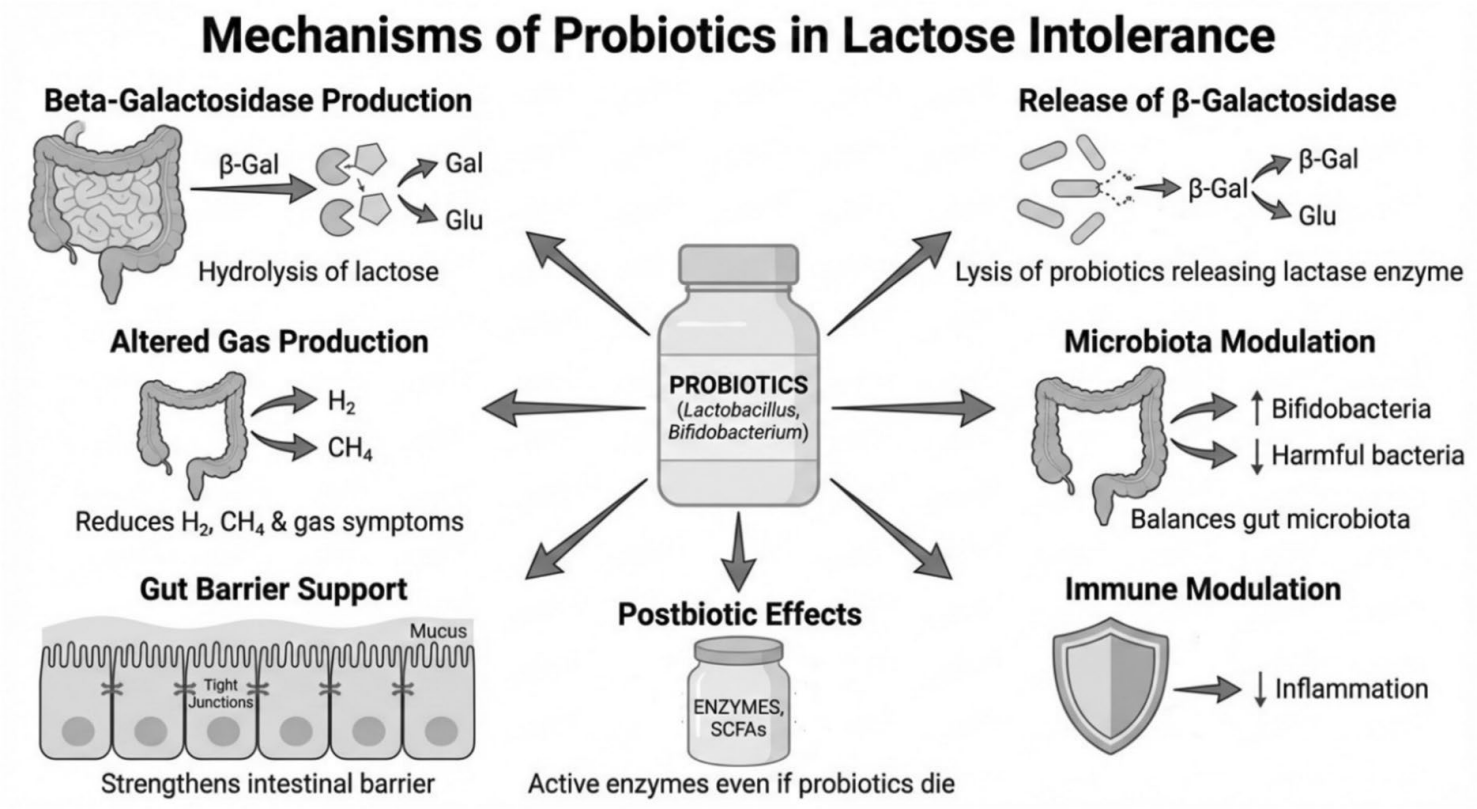
Mechanisms by which probiotics may alleviate symptoms of LI. Probiotic microorganisms may reduce LI symptoms through complementary pathways. The principal mechanism is delivery of microbial beta-galactosidase (lactase) activity to the gut lumen, promoting hydrolysis of lactose in the small intestine and thereby decreasing the amount of lactose reaching the colon. This effect may be enhanced when probiotics are consumed in fermented dairy matrices that already contain microbial beta-galactosidase. During gastrointestinal transit, probiotics may also release beta-galactosidase via cell lysis or extracellular enzyme export, supporting clinical benefit even when viability decreases (postbiotic-like enzyme activity). By reducing colonic lactose availability and altering microbial metabolism, probiotics can modulate fermentation outputs of H₂, CO₂, CH₄, H₂S and SCFAs and may suppress gas-producing microbes through competitive interactions. In parallel, probiotics may modulate the colonic microbiota increasing lactose-utilizing taxa and overall beta-galactosidase activity, while typically remaining transient rather than permanently colonizing. Finally, probiotics may improve symptom tolerance by supporting intestinal barrier function (tight-junction integrity and mucus layer) and immune modulation (reduction of pro-inflammatory signaling), contributing to overall intestinal well-being in LI

Probiotics contribute to overall intestinal well-being, which is critical in mitigating LI symptoms (Angima et al. 2024; Gingold-Belfer et al. 2020). Probiotics strength the host's intestinal barrier function by decreasing mucosal permeability and increasing intercellular integrity of apical tight junctions. For example, Bifidobacterium animalis can upregulate the expression of tight junction proteins. Lactobacillus acidophilus and Bifidobacterium longum can promote mucus secretion, improving barrier functions as well. Finally, probiotics can modulate immune responses and reduce pro-inflammatory cytokines (Fassio et al. 2018; Szilagyi and Ishayek 2018; Kurilshikov et al. 2021; Hill et al. 2014; Salminen et al. 2021). These direct and host-directed mechanisms are summarized in Fig. 1.

## Probiotics: evidence from clinical trials in LI

Randomized control trials (RCT's) and meta-analysis have demonstrated the efficacy of probiotics in reducing symptom severity scores and hydrogen excretion in breath tests. For example, a meta-analysis of 12 clinical studies investigated the efficacy of probiotic administration in mitigating symptoms of lactose intolerance in adults. Utilizing a mixed-effect model to calculate standardized mean differences (SMD), the study evaluated outcomes including abdominal pain, diarrhea, and flatulence. The results indicated that probiotic intervention significantly reduced clinical symptoms associated with lactose malabsorption. The most pronounced effect was observed in the area under the curve (AUC) of symptom severity, which demonstrated a substantial decrease (SMD = −4.96; 95% CI: −6.92 to −3.00). Subgroup analyses revealed distinct advantages based on probiotic formulation: mono-strain probiotics demonstrated superior efficacy in reducing both abdominal pain and total symptom scores, while multi-strain combinations were found to be particularly effective in the management of flatulence (Ahn et al. 2023). However, efficacy was highly dependent on specific bacterial strains, dosage, and formulation used (Leis et al. 2020; Ahn et al. 2023; Rasinkangas et al. 2022; Angima et al. 2024; Oak and Jha 2019). In one study the administration of Lactobacillus casei Shirota and Bifidobacterium breve Yakult (mix preparation) for one month led to a significant and sustained (for 3 months) improvement in symptom severity scores and in hydrogen gas production in patients with LI (Almeida et al. 2012). In another study, the administration of Bifidobacterium longum administrated in capsules which contained approximately 2 × 10^8^ colony forming units (CFU) and yogurt enriched with Bifidobacterium animalis which contained approximately 10^8^ CFU per gram for 2 weeks led to a significant improvement in symptom severity scores and to modification of the colonic microbiota (increased galactosidase activity) in patients with LI (Jaagura et al. 2022).

In a randomized trial involving forty subjects who received 8 × 10^8^ CFU/day of Lactobacillus reuteri for 10 days showed significant improvements in abdominal pain, bloating, diarrhea, and flatulence. Studies involving different strains of Lactobacillus acidophilus have yielded varied results. In one crossover trial, the DDS-1 strain of L. acidophilus (10^9^ CFU per day in capsules for four weeks) was associated with significant improvements in diarrhea, cramping, and vomiting after lactose challenge. Other trials with L. acidophilus showed a dose-dependent improvements in symptoms (up to 4 × 10^9^ CFU per day) (Pakdaman et al. 2016).

Multi-strain formulas have also been used for SI in clinical trials. A pilot study using a proprietary formula called BIO-25 over six months demonstrated symptom resolution, particularly reducing the severity of bloating and flatulence. However, BIO-25 normalized hydrogen excretion in breath tests in only 25% of patients (Gingold-Belfer et al. 2020). In another study using a combination of Bifidobacterium animalis and Lactiplantibacillus plantarum the comparison of baseline scores with those obtained after supplementation and a two-week follow-up within the groups indicated that the probiotic product significantly reduced instances of diarrhoea and flatulence (*p* < 0.05) (Roškar et al. 2017). There were no reported adverse effects, and the observed efficacy trends support the potential of the probiotic as a viable option for alleviating symptoms of lactose intolerance.

The main strength of the evidence is the consistent finding across multiple randomized controlled trials that probiotic supplementation, particularly those that possess galactosidase activity (Bifidobacterium and Lactobacillus species), reduces self-reported symptoms such as bloating, flatulence, and diarrhea, and improves objective measures like hydrogen excretion in breath tests. Furthermore, the demonstrated persistence of efficacy even months after treatment suspension suggests the potential for sustained colonic microbiota adaptation. One meta-regression analysis indicated that administering an excessively high dosage of probiotics was associated with a significant improvement in symptom scores (Vitellio et al. 2019; Salminen et al. 2021; Leis et al. 2020; Ahn et al. 2023; Rasinkangas et al. 2022; Almeida et al. 2012; Jaagura et al. 2022; Pakdaman et al. 2016; Roškar et al. 2017; Masoumi et al. 2021; Aguilera et al. 2021).

As suggested earlier, limitations include the pronounced heterogeneity across studies concerning methodology, intervention duration (ranging from a few days to six weeks or even six months), and the wide range of probiotic dosages tested (spanning 10^7^ to 10^11^ CFUs per day), making direct comparison and meta-analysis challenging (Misselwitz et al. 2019; Fassio et al. 2018; Vitellio et al. 2019; Leis et al. 2020; Ahn et al. 2023; Gingold-Belfer et al. 2020; Oak and Jha 2019; Almeida et al. 2012; Jaagura et al. 2022; Pakdaman et al. 2016; Roškar et al. 2017; Masoumi et al. 2021; Aguilera et al. 2021). In addition, and because many effects are strain-specific, there is inconsistency in the results, as demonstrated with the L. acidophilus species. Similarly, some multi-strain probiotics failed to improve symptoms in LI, highlighting that efficacy cannot be generalized across all products of probiotic class (Leis et al. 2020; Ahn et al. 2023; Oliveira et al. 2022). For example, combining Lactobacillus plantarum with Bifidobacterium animalis was ineffective for LI in one trial, but the combination of Bifidobacterium longum BB536 and Lactobacillus rhamnosus HN001 plus Vitamin B6, significantly improved bloating and constipation (Ruzsanyi et al. 2016; Vitellio et al. 2019; Ahn et al. 2023).

Overall, studies have demonstrated that probiotic supplementation, especially strains belonging to the Lactobacillus and Bifidobacterium genera were the most effective in improving LI. However, clinical evidence remains inconclusive due to lack of standardizations across clinical trials (Vitellio et al. 2019; Ahn et al. 2023; Roškar et al. 2017; Masoumi et al. 2021; Aguilera et al. 2021; Oliveira et al. 2022; Liao et al. 2022).

Synbiotics are an example of combination therapy using a mixture of probiotics and prebiotics as substances used by probiotics bacteria in a single compound. Beside their beneficially affect to the host, the addition of prebiotics improves the survival and successful colonization of the probiotic bacteria (Swanson et al. 2020). Prebiotics are typically non-digestible oligosaccharides such as fructans and galactans. Prebiotics stimulate the growth and modulate the metabolic activity of gut microbiota (adaptive—beta-galactosidase activity). Clinical trials with prebiotics such as galacto-oligosaccharides (GOS) have shown that the addition of GOS reduced LI related symptoms by increasing the concentration of lactose-fermenting bacteria like Lactobacillus and Bifidobacterium species in the colon (Savaiano et al. 2013; Angima et al. 2025). Another type of combination involves pairing acid lactase derived from Aspergillus oryzae with traditional yogurt bacteria. This combination synergistically enhances lactose digestion (Vrese et al. 2015). To summarize, although clinical evidence supports the beneficial effects of synbiotics, further research is still needed to prove synergistic effect (Leis et al. 2020; Oliveira et al. 2022; Swanson et al. 2020; Angima et al. 2025).

Probiotics, particularly strains derived from Lactobacillus and Bifidobacterium, are generally considered safe for the general population; mild transient gastrointestinal symptoms may occur, and caution is advised in severely immunocompromised individuals (Angima et al. 2024; Su et al. 2020; Markowiak and Śliżewska 2017; Sanders et al. 2019; Stadlbauer 2015; Catanzaro et al. 2021). Because efficacy is strain- and dose-specific and commercial products vary, recommendations should prioritize preparations with documented strain identity, viable counts through shelf life, and transparent labeling produced under quality-controlled manufacturing (Marinova et al. 2019; Ahire et al. 2023). Regulatory requirements differ across jurisdictions; therefore, clinicians should verify product documentation and clinical evidence when recommending probiotics for LI (Marinova et al. 2019; Ahire et al. 2023; O'Toole et al. 2017).

## Future directions and research gaps

Despite the accumulating evidence suggesting probiotics offer an overall positive benefit for LI, several significant gaps in clinical research must be addressed to standardize this therapeutic approach. A major limitation is the wide heterogeneity across studies, encompassing differences in methodology, duration of intervention, and the range of probiotic dosages tested, which complicates direct comparisons and meta-analysis (Suez et al. 2019; Zmora et al. 2018; Sarita et al. 2025). As mentioned, efficacy is fundamentally strain-specific, so further studies are clearly required to provide high-quality comparative data on the efficacy of different strains and strategies (Zmora et al. 2018; Sarita et al. 2025). In addition, novel strategies to measure strains concentrations and preparations must be developed. Another important gap is the unknown long-time effects of probiotics, as most published clinical trials last between 2 weeks to 3 months (O'Toole et al. 2017; Suez et al. 2019; Sarita et al. 2025). Thus, clinical trial must assess the long-term efficacy as LI is a chronic condition and it crucial to understand whether effects may wane after cessation or not. Research should also evaluate inter-individual variability (e.g., baseline microbiota, IBS overlap, and visceral sensitivity) and identify responder phenotypes for specific strains. Researchers need to investigate which specific probiotic strains could elicit benefit for defined patient populations (Singh and Natraj 2021; Montazeri-Najafabady 2025; Anguita-Ruiz et al. 2020; Szilagyi et al. 2010; Foo et al. 2017; Inda et al. 2019).

Recent work also highlights that lactose intolerance symptoms reflect both lactase nonpersistence (malabsorption) and host sensitivity, with inter-individual variability shaped by diet, genetics, and the gut microbiome. Large cohort studies link lactase-persistence variants to higher Bifidobacterium abundance and distinct microbiome patterns relevant to dairy tolerance (Kato et al. 2018; Schmidt et al. 2020). Systematic reviews and meta-analyses continue to refine the evidence base for probiotic interventions, underscoring strain specificity and heterogeneity across products and trial designs (Leis et al. 2020; Ahn et al. 2023; Oliveira et al. 2022). Dietary approaches that incorporate fermented dairy or fortified products (e.g., probiotic yogurts/ice creams) may improve tolerance while supporting adherence to nutrient recommendations (Angima et al. 2024; Masoumi et al. 2021; Aguilera et al. 2021; Oliveira et al. 2022). Guidelines and consensus documents emphasize standardized definitions (probiotic/synbiotic/postbiotic), safety, and rigorous reporting (e.g., PRISMA) for interpreting clinical evidence and translating findings into practice (Ianiro et al. 2016; Gibson et al. 2017; Salminen et al. 2021; Pakdaman et al. 2016; Su et al. 2020; Markowiak and Śliżewska 2017; Sanders et al. 2019; O'Toole et al. 2017; Suez et al. 2019; Zmora et al. 2018; Sarita et al. 2025). Recent host-genetics studies underscore that the lactase locus is a reproducible determinant of Bifidobacterium abundance and other microbiome features, supporting a gene, diet and microbiome framework for symptom variability (Kurilshikov et al. 2021; Vitellio et al. 2019; Salminen et al. 2021; Sanders et al. 2019; Zmora et al. 2018; Foo et al. 2017; Inda et al. 2019). More broadly, contemporary discussions emphasize that probiotic effects can be context-dependent, with variable colonization resistance and unanswered questions about mechanisms, necessitating careful product selection and monitoring (O'Toole et al. 2017; Suez et al. 2019; Zmora et al. 2018; Sarita et al. 2025; Foo et al. 2017; Inda et al. 2019). Enzyme-based strategies (lactase) remain an important comparator in clinical practice and trials (Fassio et al. 2018; Zmora et al. 2018), and the distinction between lactose malabsorption and lactose sensitivity can help interpret discordant tests and symptoms (Fassio et al. 2018; Foo et al. 2017; Inda et al. 2019).

Looking to the future, the advent of next-generation probiotics and live biotherapeutics, combined with precision medicine approaches, holds promise for creating optimized formulations for LI. Future products may focus on strains exhibiting high beta-galactosidase activity and strong gastrointestinal resilience, while also being customized to align with individual host characteristics and microbiome traits, such as colonization resistance and baseline gas production profiles (Singh and Natraj 2021; Montazeri-Najafabady 2025; Foo et al. 2017; Inda et al. 2019).

## Conclusion

This review of the current literature confirms that lactose intolerance, arising from lactase enzyme deficiency, presents a substantial challenge to the patient's quality of life and nutrition. Traditional management, centered on dietary restriction, carries the serious risk of nutrient deficiencies, particularly affecting calcium and vitamin D intake (Misselwitz et al. 2019; Fassio et al. 2018; Bouchoucha et al. 2021; Hodges et al. 2019). However, accumulating clinical evidence showed that probiotic supplementation improves symptoms and hydrogen excretion in patients suffering from LI (Table 1; Vitellio et al. 2019; Ahn et al. 2023; Rasinkangas et al. 2022; Almeida et al. 2012; Jaagura et al. 2022; Pakdaman et al. 2016; Roškar et al. 2017; Masoumi et al. 2021; Aguilera et al. 2021; Oliveira et al. 2022). As visually summarized in Fig. 1, the efficacy of this approach is largely attributed to the delivery of microbial beta-galactosidase activity in strains like Lactobacillus and Bifidobacterium. This leads to direct hydrolysis of lactose in the small intestine and favorable modulation of the colonic microbiota toward improved lactose adaptation, effectively interrupting the pathophysiological cascade that causes symptoms (Vitellio et al. 2019; Leis et al. 2020; Rasinkangas et al. 2022; Angima et al. 2024; Gingold-Belfer et al. 2020; Masoumi et al. 2021; Aguilera et al. 2021; Oliveira et al. 2022; Liao et al. 2022). In some studies, it was observed that these beneficial effects persist for 3 months after cessation of supplementation. Thus, probiotics may represent adjunct therapy for LI management without using exogenous lactase supplements or consuming lactose-reduced products (Vitellio et al. 2019; Masoumi et al. 2021; Aguilera et al. 2021; Oliveira et al. 2022; Liao et al. 2022; Su et al. 2020; Markowiak and Śliżewska 2017; Sanders et al. 2019; O'Toole et al. 2017; Foo et al. 2017; Inda et al. 2019). Moving forward, robust research focused on strain specificity, dosage optimization, and long-term trials must continue to standardize this intervention (Fassio et al. 2018; Singh and Natraj 2021; Montazeri-Najafabady 2025; Anguita-Ruiz et al. 2020; Szilagyi et al. 2010; Foo et al. 2017; Inda et al. 2019). Successful outcomes demonstrate the clear potential integration of probiotics into definitive dietary management guidelines to help patients increase their consumption of beneficial milk and dairy products, thereby improving overall nutritional status without incurring the chronic discomfort associated with lactose malabsorption (Fassio et al. 2018; Singh and Natraj 2021; Montazeri-Najafabady 2025; Anguita-Ruiz et al. 2020; Szilagyi et al. 2010; Foo et al. 2017; Inda et al. 2019).

## Data Availability

No datasets were generated or analysed during the current study.

## References

[CR1] Aguilera G, Cárcamo C, Soto-Alarcón S, Gotteland M (2021) Improvement in lactose tolerance in hypolactasic subjects consuming ice creams with high or low concentrations of *Bifidobacterium bifidum* 900791. Foods 10(10):2468. 10.3390/foods10102468. (**Published 2021 Oct 15**) 34681517 10.3390/foods10102468 PMC8535838

[CR2] Ahire JJ, Rohilla A, Kumar V, Tiwari A (2023) Quality management of probiotics: ensuring safety and maximizing health benefits. Curr Microbiol 81(1):1. 10.1007/s00284-023-03526-3. (**Published 2023 Nov 8**) 37935938 10.1007/s00284-023-03526-3

[CR3] Ahn SI, Kim MS, Park DG, Han BK, Kim YJ (2023) Effects of probiotics administration on lactose intolerance in adulthood: A meta-analysis. J Dairy Sci 106(7):4489–4501. 10.3168/jds.2022-22762 37225575 10.3168/jds.2022-22762

[CR4] Almeida CC, Lorena SL, Pavan CR, Akasaka HM, Mesquita MA (2012) Beneficial effects of long-term consumption of a probiotic combination of *Lactobacillus casei* Shirota and *Bifidobacterium breve* Yakult may persist after suspension of therapy in lactose-intolerant patients. Nutr Clin Pract 27(2):247–251. 10.1177/0884533612440289 22402407 10.1177/0884533612440289

[CR5] Angima G, Qu Y, Park SH, Dallas DC (2024) Prebiotic strategies to manage lactose intolerance symptoms. Nutrients 16(7):1002. 10.3390/nu16071002. (**Published 2024 Mar 29**) 38613035 10.3390/nu16071002 PMC11013211

[CR6] Angima G, Qu Y, Kim E, Bobe G, Dallas DC, Park SH (2025) Effects of galactooligosaccharides (GOS) on the gut microbiota in lactose intolerant individuals. LWT 216:117291. 10.1016/j.lwt.2024.117291

[CR7] Anguita-Ruiz A, Aguilera CM, Gil Á (2020) Genetics of lactose intolerance: an updated review and online interactive world maps of phenotype and genotype frequencies. Nutrients 12(9):2689. 10.3390/nu12092689. (**Published 2020 Sep 3**) 32899182 10.3390/nu12092689 PMC7551416

[CR8] Baijal R, Tandon RK (2020) Effect of lactase on symptoms and hydrogen breath levels in lactose intolerance: A crossover placebo-controlled study. JGH Open 5(1):143–148. 10.1002/jgh3.12463. (**Published 2020 Dec 1**) 33490624 10.1002/jgh3.12463 PMC7812489

[CR9] Berstad A, Raa J, Midtvedt T, Valeur J (2016) Probiotic lactic acid bacteria - the fledgling cuckoos of the gut? Microb Ecol Health Dis 27(0):31557. 10.3402/mehd.v27.31557 27235098 10.3402/mehd.v27.31557 PMC4884264

[CR10] Binder HJ (2010) Role of colonic short-chain fatty acid transport in diarrhea. Annu Rev Physiol 72:297–313. 10.1146/annurev-physiol-021909-135817 20148677 10.1146/annurev-physiol-021909-135817

[CR11] Bonder MJ, Kurilshikov A, Tigchelaar EF et al (2016) The effect of host genetics on the gut microbiome. Nat Genet 48(11):1407–1412. 10.1038/ng.3663 27694959 10.1038/ng.3663

[CR12] Bouchoucha M, Fysekidis M, Rompteaux P, Raynaud JJ, Sabate JM, Benamouzig R (2021) Lactose sensitivity and lactose malabsorption: the 2 faces of lactose intolerance. J Neurogastroenterol Motil 27(2):257–264. 10.5056/jnm20094 33361550 10.5056/jnm20094 PMC8026364

[CR13] Campbell AK, Matthews SB, Vassel N et al (2010) Bacterial metabolic “toxins”: a new mechanism for lactose and food intolerance, and irritable bowel syndrome. Toxicology 278(3):268–276. 10.1016/j.tox.2010.09.001 20851732 10.1016/j.tox.2010.09.001

[CR14] Catanzaro R, Sciuto M, Marotta F (2021) Lactose intolerance: an update on its pathogenesis, diagnosis, and treatment. Nutr Res 89:23–34. 10.1016/j.nutres.2021.02.003 33887513 10.1016/j.nutres.2021.02.003

[CR15] de Vrese M, Laue C, Offick B et al (2015) A combination of acid lactase from *Aspergillus oryzae* and yogurt bacteria improves lactose digestion in lactose maldigesters synergistically: a randomized, controlled, double-blind cross-over trial. Clin Nutr 34(3):394–399. 10.1016/j.clnu.2014.06.012 25042846 10.1016/j.clnu.2014.06.012

[CR16] Deng Y, Misselwitz B, Dai N, Fox M (2015) Lactose intolerance in adults: Biological mechanism and dietary management. Nutrients 7(9):8020–8035. 10.3390/nu7095380. (**Published 2015 Sep 18**) 26393648 10.3390/nu7095380 PMC4586575

[CR17] Eadala P, Waud JP, Matthews SB, Green JT, Campbell AK (2009) Quantifying the “hidden” lactose in drugs used for the treatment of gastrointestinal conditions. Aliment Pharmacol Ther 29(6):677–687. 10.1111/j.1365-2036.2008.03889.x 19035974 10.1111/j.1365-2036.2008.03889.x

[CR18] Fassio F, Facioni MS, Guagnini F (2018) Lactose maldigestion, malabsorption, and intolerance: A comprehensive review with a focus on current management and future perspectives. Nutrients 10(11):1599. 10.3390/nu10111599. (**Published 2018 Nov 1**) 30388735 10.3390/nu10111599 PMC6265758

[CR19] Foo JL, Ling H, Lee YS, Chang MW (2017) Microbiome engineering: current applications and its future. Biotechnol J 12(3):10.1002/biot.201600099. 10.1002/biot.201600099 10.1002/biot.201600099 28133942

[CR20] Gibson GR, Hutkins R, Sanders ME et al (2017) Expert consensus document: The International Scientific Association for Probiotics and Prebiotics (ISAPP) consensus statement on the definition and scope of prebiotics. Nat Rev Gastroenterol Hepatol 14(8):491–502. 10.1038/nrgastro.2017.75 28611480 10.1038/nrgastro.2017.75

[CR21] Gingold-Belfer R, Levy S, Layfer O et al (2020) Use of a novel probiotic formulation to alleviate lactose intolerance symptoms-a pilot study. Probiotics Antimicrob Proteins 12(1):112–118. 10.1007/s12602-018-9507-7 30617948 10.1007/s12602-018-9507-7

[CR22] Hammer HF, Fox MR, Keller J et al (2022) European guideline on indications, performance, and clinical impact of hydrogen and methane breath tests in adult and pediatric patients: European Association for Gastroenterology, Endoscopy and Nutrition, European Society of Neurogastroenterology and Motility, and European Society for Paediatric Gastroenterology Hepatology and Nutrition consensus. United Eur Gastroenterol J 10(1):15–40. 10.1002/ueg2.12133 10.1002/ueg2.12133 PMC8830282 34431620

[CR23] Harvey L, Ludwig T, Hou AQ et al (2018) Prevalence, cause and diagnosis of lactose intolerance in children aged 1–5 years: a systematic review of 1995–2015 literature. Asia Pac J Clin Nutr 27(1):29–46. 10.6133/apjcn.022017.05 29222879 10.6133/apjcn.022017.05

[CR24] Hill C, Guarner F, Reid G et al (2014) Expert consensus document. The International Scientific Association for Probiotics and Prebiotics consensus statement on the scope and appropriate use of the term probiotic. Nat Rev Gastroenterol Hepatol 11(8):506–514. 10.1038/nrgastro.2014.66 24912386 10.1038/nrgastro.2014.66

[CR25] Hodges JK, Cao S, Cladis DP, Weaver CM (2019) Lactose intolerance and bone health: The challenge of ensuring adequate calcium intake. Nutrients 11(4):718. 10.3390/nu11040718. (**Published 2019 Mar 28**) 30925689 10.3390/nu11040718 PMC6521087

[CR26] Ianiro G, Pecere S, Giorgio V, Gasbarrini A, Cammarota G (2016) Digestive enzyme supplementation in gastrointestinal diseases. Curr Drug Metab 17(2):187–193. 10.2174/138920021702160114150137 26806042 10.2174/138920021702160114150137 PMC4923703

[CR27] Ibba I, Gilli A, Boi MF, Usai P (2014) Effects of exogenous lactase administration on hydrogen breath excretion and intestinal symptoms in patients presenting lactose malabsorption and intolerance. Biomed Res Int 2014:680196. 10.1155/2014/680196 24967391 10.1155/2014/680196 PMC4055537

[CR28] Inda ME, Broset E, Lu TK, de la Fuente-Nunez C (2019) Emerging frontiers in microbiome engineering. Trends Immunol 40(10):952–973. 10.1016/j.it.2019.08.007 31601521 10.1016/j.it.2019.08.007

[CR29] Jaagura M, Part N, Adamberg K, Kazantseva J, Viiard E (2022) Consumption of multi-fiber enriched yogurt is associated with increase of *Bifidobacterium animalis* and butyrate producing bacteria in human fecal microbiota. J Funct Foods 88:104899. 10.1016/j.jff.2021.104899

[CR30] JanssenDuijghuijsen L, Looijesteijn E, van den Belt M et al (2024) Changes in gut microbiota and lactose intolerance symptoms before and after daily lactose supplementation in individuals with the lactase nonpersistent genotype. Am J Clin Nutr 119(3):702–710. 10.1016/j.ajcnut.2023.12.016 38159728 10.1016/j.ajcnut.2023.12.016

[CR31] Kato K, Ishida S, Tanaka M, Mitsuyama E, Xiao JZ, Odamaki T (2018) Association between functional lactase variants and a high abundance of *Bifidobacterium* in the gut of healthy Japanese people. PLoS ONE 13(10):e0206189. 10.1371/journal.pone.0206189. (**Published 2018 Oct 19**) 30339693 10.1371/journal.pone.0206189 PMC6195297

[CR32] Kurilshikov A, Medina-Gomez C, Bacigalupe R et al (2021) Large-scale association analyses identify host factors influencing human gut microbiome composition. Nat Genet 53(2):156–165. 10.1038/s41588-020-00763-1 33462485 10.1038/s41588-020-00763-1 PMC8515199

[CR33] Leis R, de Castro MJ, de Lamas C, Picáns R, Couce ML (2020) Effects of prebiotic and probiotic supplementation on lactase deficiency and lactose intolerance: a systematic review of controlled trials. Nutrients 12(5):1487. 10.3390/nu12051487. (**Published 2020 May 20**) 32443748 10.3390/nu12051487 PMC7284493

[CR34] Liao W, Su M, Zhang D (2022) A study on the effect of symbiotic fermented milk products on human gastrointestinal health: double-blind randomized controlled clinical trial. Food Sci Nutr 10(9):2947–2955. 10.1002/fsn3.2890. (**Published 2022 May 3**) 36171774 10.1002/fsn3.2890 PMC9469858

[CR35] Marinova VY, Rasheva IK, Kizheva YK, Dermenzhieva YD, Hristova PK (2019) Microbiological quality of probiotic dietary supplements. Biotechnol Biotechnol Equip 33(1):834–841. 10.1080/13102818.2019.1621208

[CR36] Markowiak P, Śliżewska K (2017) Effects of probiotics, prebiotics, and synbiotics on human health. Nutrients 9(9):1021. 10.3390/nu9091021. (**Published 2017 Sep 15**) 28914794 10.3390/nu9091021 PMC5622781

[CR37] Masoumi SJ, Mehrabani D, Saberifiroozi M, Fattahi MR, Moradi F, Najafi M (2021) The effect of yogurt fortified with *Lactobacillus acidophilus* and *Bifidobacterium* sp. probiotic in patients with lactose intolerance. Food Sci Nutr 9(3):1704–1711. 10.1002/fsn3.2145. (**Published 2021 Jan 20**) 33747481 10.1002/fsn3.2145 PMC7958570

[CR38] Misselwitz B, Butter M, Verbeke K, Fox MR (2019) Update on lactose malabsorption and intolerance: pathogenesis, diagnosis and clinical management. Gut 68(11):2080–2091. 10.1136/gutjnl-2019-318404 31427404 10.1136/gutjnl-2019-318404 PMC6839734

[CR39] Montalto M, Nucera G, Santoro L et al (2005) &lt;article-title update="added"&gt;effect of exogenous β-galactosidase in patients with lactose malabsorption and intolerance: a crossover double-blind placebo-controlled study. Eur J Clin Nutr 59(4):489–493. 10.1038/sj.ejcn.1602098 15674309 10.1038/sj.ejcn.1602098

[CR40] Montazeri-Najafabady N. From One-Size-Fits-All to Precision Medicine: The Promise of Personalized Probiotics. Probiotics Antimicrob Proteins. Published online October 29, 2025. 10.1007/s12602-025-10815-9 10.1007/s12602-025-10815-9 41160388

[CR41] Oak SJ, Jha R (2019) The effects of probiotics in lactose intolerance: A systematic review. Crit Rev Food Sci Nutr 59(11):1675–1683. 10.1080/10408398.2018.1425977 29425071 10.1080/10408398.2018.1425977

[CR42] Oliveira LS, Wendt GW, Crestani APJ, Casaril KBPB (2022) The use of probiotics and prebiotics can enable the ingestion of dairy products by lactose intolerant individuals. Clin Nutr 41(12):2644–2650. 10.1016/j.clnu.2022.10.003 36308983 10.1016/j.clnu.2022.10.003

[CR43] O’Toole PW, Marchesi JR, Hill C (2017) Next-generation probiotics: the spectrum from probiotics to live biotherapeutics. Nat Microbiol 2(5):17057. 10.1038/nmicrobiol.2017.57 28440276 10.1038/nmicrobiol.2017.57

[CR44] Pakdaman MN, Udani JK, Molina JP, Shahani M (2016) The effects of the DDS-1 strain of *Lactobacillus* on symptomatic relief for lactose intolerance - a randomized, double-blind, placebo-controlled, crossover clinical trial. Nutr J 15(1):56. 10.1186/s12937-016-0172-y. (**Published 2016 May 20**) 27207411 10.1186/s12937-016-0172-y PMC4875742

[CR45] Rasinkangas P, Forssten SD, Marttinen M et al (2022) *Bifidobacterium animalis* subsp. *lactis* Bi-07 supports lactose digestion in vitro and in randomized, placebo- and lactase-controlled clinical trials. Am J Clin Nutr 116(6):1580–1594. 10.1093/ajcn/nqac264 36149331 10.1093/ajcn/nqac264 PMC9761758

[CR46] Read NW (1982) Diarrhoea: the failure of colonic salvage. Lancet 2(8296):481–483. 10.1016/s0140-6736(82)90504-9 6125648 10.1016/s0140-6736(82)90504-9

[CR47] Rezaie A, Buresi M, Lembo A et al (2017) Hydrogen and methane-based breath testing in gastrointestinal disorders: The North American Consensus. Am J Gastroenterol 112(5):775–784. 10.1038/ajg.2017.46 28323273 10.1038/ajg.2017.46 PMC5418558

[CR48] Roškar I, Švigelj K, Štempelj M et al (2017) Effects of a probiotic combination (Bifidobacterium animalis IM386 and Lactiplantibacillus plantarum MP2026) on lactose intolerance symptoms: a randomized, double-blind, placebo-controlled trial. Nutrients 9(10):1105. 10.3390/nu9101105 28994714 10.3390/nu9101105 PMC5691721

[CR49] Ruzsanyi V, Heinz-Erian P, Entenmann A et al (2016) Diagnosing lactose malabsorption in children: difficulties in interpreting hydrogen breath test results. J Breath Res 10(1):016015. 10.1088/1752-7155/10/1/016015. (**Published 2016 Mar 2**) 26934035 10.1088/1752-7155/10/1/016015

[CR50] Salminen S, Collado MC, Endo A, et al. The International Scientific Association of Probiotics and Prebiotics (ISAPP) consensus statement on the definition and scope of postbiotics. Nat Rev Gastroenterol Hepatol. 2021;18(9):649-667. 10.1038/s41575-021-00440-6 10.1038/s41575-021-00440-6 PMC8387231 33948025

[CR51] Sanders ME, Merenstein DJ, Reid G, Gibson GR, Rastall RA (2019) Probiotics and prebiotics in intestinal health and disease: from biology to the clinic. Nat Rev Gastroenterol Hepatol 16(10):605–616. 10.1038/s41575-019-0173-3 31296969 10.1038/s41575-019-0173-3

[CR52] Sarita B, Samadhan D, Hassan MZ, Kovaleva EG (2025) A comprehensive review of probiotics and human health-current prospective and applications. Front Microbiol 15:1487641. 10.3389/fmicb.2024.1487641 39834364 10.3389/fmicb.2024.1487641 PMC11743475

[CR53] Savaiano DA, Hutkins RW (2021) Yogurt, cultured fermented milk, and health: a systematic review. Nutr Rev 79(5):599–614. 10.1093/nutrit/nuaa013 32447398 10.1093/nutrit/nuaa013 PMC8579104

[CR54] Savaiano DA, Ritter AJ, Klaenhammer TR et al (2013) Improving lactose digestion and symptoms of lactose intolerance with a novel galacto-oligosaccharide (RP-G28): a randomized, double-blind clinical trial. Nutr J 12(1):160. 10.1186/1475-2891-12-160 24330605 10.1186/1475-2891-12-160 PMC3878758

[CR55] Schmidt V, Enav H, Spector TD, Youngblut ND, Ley RE (2020) Strain-level analysis of *Bifidobacterium* spp. from gut microbiomes of adults with differing lactase persistence genotypes. mSystems 5(5):e00911-20. 10.1128/mSystems.00911-20. (**Published 2020 Sep 29**) 32994293 10.1128/mSystems.00911-20 PMC7527142

[CR56] Singh TP, Natraj BH (2021) Next-generation probiotics: a promising approach towards designing personalized medicine. Crit Rev Microbiol 47(4):479–498. 10.1080/1040841X.2021.1902940 33822669 10.1080/1040841X.2021.1902940

[CR57] Stadlbauer V (2015) Immunosuppression and probiotics: are they effective and safe? Benef Microbes 6(6):823–828. 10.3920/BM2015.0065 26287986 10.3920/BM2015.0065

[CR58] Su GL, Ko CW, Bercik P et al (2020) AGA clinical practice guidelines on the role of probiotics in the management of gastrointestinal disorders. Gastroenterology 159(2):697–705. 10.1053/j.gastro.2020.05.059 32531291 10.1053/j.gastro.2020.05.059

[CR59] Suez J, Zmora N, Segal E, Elinav E (2019) The pros, cons, and many unknowns of probiotics. Nat Med 25(5):716–729. 10.1038/s41591-019-0439-x 31061539 10.1038/s41591-019-0439-x

[CR60] Swanson KS, Gibson GR, Hutkins R, et al. The International Scientific Association for Probiotics and Prebiotics (ISAPP) consensus statement on the definition and scope of synbiotics. Nat Rev Gastroenterol Hepatol. 2020;17(11):687-701. 10.1038/s41575-020-0344-2 10.1038/s41575-020-0344-2 PMC7581511 32826966

[CR61] Szilagyi A, Ishayek N (2018) Lactose intolerance, dairy avoidance, and treatment options. Nutrients 10(12):1994. 10.3390/nu10121994. (**Published 2018 Dec 15**) 30558337 10.3390/nu10121994 PMC6316316

[CR62] Szilagyi A, Shrier I, Heilpern D et al (2010) Differential impact of lactose/lactase phenotype on colonic microflora. Can J Gastroenterol 24(6):373–379. 10.1155/2010/649312 20559580 10.1155/2010/649312 PMC2898492

[CR63] Vesa TH, Marteau P, Korpela R (2000) Lactose intolerance. J Am Coll Nutr 19(2 Suppl):165S-175S. 10.1080/07315724.2000.10718086 10759141 10.1080/07315724.2000.10718086

[CR64] Vitellio P, Celano G, Bonfrate L, Gobbetti M, Portincasa P, De Angelis M (2019) Effects of *Bifidobacterium longum* and *Lactobacillus rhamnosus* on gut microbiota in patients with lactose intolerance and persisting functional gastrointestinal symptoms: A randomised, double-blind, cross-over study. Nutrients 11(4):886. 10.3390/nu11040886. (**Published 2019 Apr 19**) 31010241 10.3390/nu11040886 PMC6520754

[CR65] Zmora N, Zilberman-Schapira G, Suez J et al (2018) Personalized gut mucosal colonization resistance to empiric probiotics is associated with unique host and microbiome features. Cell 174(6):1388-1405.e21. 10.1016/j.cell.2018.08.041 30193112 10.1016/j.cell.2018.08.041

